# Diagnosis and classification of portosystemic shunts: a machine learning retrospective case-control study

**DOI:** 10.3389/fvets.2024.1291318

**Published:** 2024-04-04

**Authors:** Makan Farhoodimoghadam, Krystle L. Reagan, Allison L. Zwingenberger

**Affiliations:** ^1^Department of Computer Science, University of California, Davis, Davis, CA, United States; ^2^Department of Veterinary Medicine and Epidemiology, School of Veterinary Medicine, University of California, Davis, Davis, CA, United States; ^3^Department of Surgical and Radiological Sciences, School of Veterinary Medicine, University of California, Davis, Davis, CA, United States

**Keywords:** liver disease, veterinary, artificial intelligence, hepatopathy, congenital, microvascular dysplasia

## Abstract

Diagnosis of portosystemic shunts (PSS) in dogs often requires multiple diagnostic tests, and available clinicopathologic tests have limitations in sensitivity and specificity. The objective of this study was to train and validate a machine learning model (MLM) that can accurately predict the presence of a PSS utilizing routinely collected demographic data and clinicopathologic features. Dogs diagnosed with PSS or control dogs tested for PSS but had the condition ruled out (non-PSS) were identified. Dogs were included if a complete blood count and serum chemistry panel were available from PSS diagnostic testing. Dogs with a PSS were subcategorized as having a single intrahepatic PSS, a single extrahepatic PSS, or multiple extrahepatic PSS. An extreme gradient boosting (XGboost) MLM was trained with data from 70% of the cases, and MLM performance was determined on the test set, comprising the remaining 30% of the case data. Two MLMs were created. The first was designed to predict the presence of any PSS (PSS MLM), and the second to predict the PSS subcategory (PSS SubCat MLM). The trained PSS MLM had a sensitivity of 94.3% (95% CI 90.1–96.8%) and specificity of 90.5% (95% CI 85.32–94.0%) for dogs in the test set. The area under the receiver operator characteristic curve (AUC) was 0.976 (95% CI; 0.964–0.989). The mean corpuscular hemoglobin, lymphocyte count, and serum globulin concentration were most important in prediction classification. The PSS SubCat MLM had an accuracy of 85.7% in determining the subtype of PSS of dogs in the test set, with variable sensitivity and specificity depending on PSS subtype. These MLMs have a high accuracy for diagnosing PSS; however, the prediction of PSS subclassification is less accurate. The MLMs can be used as a screening tool to increase or decrease the index of suspicion for PSS before confirmatory diagnostics such as advanced imaging are pursued.

## Introduction

Portosystemic shunts (PSS) are vascular anomalies that allow blood within the portal circulation to bypass the liver and enter the systemic circulation. This results in the clinical syndrome of hepatic encephalopathy, reduced metabolization of hepatically cleared medications, and accumulation of blood ammonia that can result in urate urinary calculi. The clinical presentation of dogs with PSS is variable and can range from subclinical to a severe, life-threatening condition. The overall incidence of congenital PSS is 0.18% (1). However, some breeds are overrepresented, with around 3% of Havanese and Yorkshire terriers being diagnosed with the condition (1–3). These vascular anomalies are subcategorized as congenital due to inappropriate attenuation of fetal vasculature or acquired when they arise due to portal hypertension. Congenital PSS are further subclassified into extrahepatic PSS, more commonly diagnosed in toy breed dogs, and intrahepatic PSS, more commonly diagnosed in large breed dogs (4, 5).

The clinical presentation of dogs with PSS can be vague and variable, with signs including failure to thrive, gastrointestinal signs, or neurologic signs (6, 7). Clinical signs could easily be attributed to other disease processes, making recognizing this condition challenging in some cases. Other hepatic diseases, including portal venous hypoplasia (PVH) and chronic hepatitis, can mimic the clinical signs and clinicopathologic findings often associated with PSS, such as anemia, decreased blood urea nitrogen concentrations, elevated liver enzymes, hypoalbuminemia, and hypoglycemia (5, 7–10).

Diagnostic tools to discriminate between PSS and other disease conditions include measurement of paired fasting and post-prandial serum bile acid concentrations and abdominal imaging studies. Elevated serum bile acid concentrations are a sensitive marker for hepatic insufficiency but cannot differentiate between PSS and other pathology, such as PVH (11). Ultrasonographic identification of PSS requires advanced technical training, and sensitivity depends on the operator’s skill level (12, 13). Additional imaging tests such as nuclear scintigraphy and computed tomography may be needed to confirm or exclude the presence of a shunt, and dogs often undergo a series of tests for a final diagnosis. Other biomarkers have been investigated to help discriminate between PSS and other liver pathologies, including serum ammonia concentrations protein C; however, these assays lack specificity for diagnosing a PSS (14, 15).

Machine learning techniques have been increasingly utilized to enhance diagnostic and predictive capabilities in the veterinary field. Some previous applications of machine learning in veterinary medicine include the diagnosis of hypoadrenocorticism, leptospirosis, and babesiosis in dogs, as well as lameness in cows (16–19). Predictable patterns in clinicopathologic data have been previously observed in dogs with PSS (6, 7, 20). This study aimed to evaluate a gradient-boosted tree machine learning model (MLM) as a predictive tool to detect PSS in dogs utilizing demographic features and features from routinely conducted blood work, the complete blood count and serum biochemistry panel. We hypothesize that MLMs when trained with clinicopathologic data from dogs with PSS or dogs tested for PSS that had the disease ruled out, can accurately discriminate between dogs that have a PSS and those that have non-PSS disease. Further, we hypothesize that MLMs can accurately classify dogs based on the subtype of PSS.

## Materials and methods

### Patient selection and ground truth determination

A retrospective case–control study, selecting dogs from the electronic medical record system of the University of California-Davis Veterinary Medical Teaching Hospital (VMTH) was performed. Records were searched from 2000 to 2020 to identify potential cases (PSS group) and controls (non-PSS group).

Dogs with PSS were identified by (1) searching for “shunt” or “PSS” in the clinical diagnosis field (2) searching for dogs with advanced imaging procedures of the abdomen (contrast CT scan, dual-phase contrast CT scan, nuclear scintigraphy of the liver) or (3) searching for dogs that underwent a PSS related surgical procedure (percutaneous intrahepatic shunt coil embolization procedure, intrahepatic shunt surgical repair, multiple or single extrahepatic shunt attenuation, or laparoscopic liver biopsy). After a medical record review (AZ), dogs were included as cases if PSS were identified on ≥1 imaging modality or during surgical intervention. If conflicting results were obtained regarding the presence of a PSS on multiple imaging modalities, the dog was excluded. Dogs were subcategorized in this group based on imaging into three groups; single intrahepatic portosystemic shunt, single extrahepatic portosystemic shunt, or multiple extrahepatic shunts based on imaging studies or surgical exploration results based on a wholistic medical record review (AZ).

Approximately equal number of dogs with non-PSS disease were identified as controls by searching for dogs that had post-prandial serum bile acid concentrations measured (Veterinary Diagnostic Laboratory, University of California-Davis). Medical records were reviewed by a single author (AZ), and dogs were included if a PSS was not identified on at least one of abdominal ultrasound, contrast CT scan, dual-phase contrast CT scan, or transrectal nuclear scintigraphy of the liver. The clinical diagnosis of the non-PSS dogs was reviewed by a single author (KR), and the primary clinical diagnosis was categorized into the following categories: gastrointestinal (including non-PSS hepatic or pancreatic disease), cardiac, immune-mediated, genitourinary, neurologic, respiratory, infectious, neoplasia, trauma and primary surgical, or undetermined.

To be included, PSS and non-PSS dogs had to have a complete blood count (Advia 120; Siemens) and serum chemistry panel (Hitachi 917 years 2000–2009; Cobas c501c/6,000 years 2009–2020) performed (Veterinary Diagnostic Laboratory, University of California-Davis) contemporaneously with the diagnostic investigation of PSS and before surgical intervention for PSS attenuation for dogs with congenital PSS. Medical records for PSS and non-PSS dogs were reviewed, and breed, sex, age (days), weight (kg), results of complete blood count, and serum biochemistry at the time of diagnosis at the VMTH were recorded. Results of abdominal imaging, surgical interventions performed, and results of liver histopathology were also recorded.

### Feature preprocessing

Serum bilirubin and gamma-glutamyltransferase have a lower level of detection >0. Values below the detection limit were recorded at 0.01 below the lower end of assay detection. Dog breeds were grouped into toy, herding, hound, non-sporting, sporting, terrier, working, foundation stock service, mix breed, or other. One-hot encoding was utilized for categorical variables.

### Machine learning model training

Classification models were trained with patient features and clinicopathologic parameters (Table 1). The data set was randomly split into a 70% training set and a 30% test set and two models were trained, one that predicts the presence of any PSS (PSS MLM) and a second that predicts the subclassification of PSS (PSS SubCat MLM). First, a gradient boosted tree (xgboost 1.7.6, Python3) was trained to discriminate between PSS and non-PSS dogs (PSS MLM) (21). The model was trained using 10-fold cross-validation, and hyperparameters were tuned using a Bayesian search algorithm optimized based on AUC-ROC. The tuned hyperparameters included gamma, learning rate, maximum depth, the number of estimators, regularization alpha, and regularization lambda (Supplementary Table S1). A second gradient boosted tree (PSS SubCat MLM) was trained to classify animals into the following categories: single intrahepatic portosystemic shunt, single extrahepatic portosystemic shunt, multiple extrahepatic shunts, or non-PSS disease. Due to class imbalance, the synthetic minority over-sampling technique (SMOTE) from imblearn (0.10.1) library was applied to the PSS SubCat MLM training data set (22, 23). Model code utilized to build these models is publicly available (https://github.com/MakanFar/pss_classification).

**Table 1:** Model features utilized to predict presence of portosystemic shunt.

Demographics	Complete blood count	Serum chemistry
Breed group	Hematocrit (%)	Anion gap (mmol/L)
Weight (kg)	Red blood cells (/𝜇L)	Sodium (mmol/L)
Age (years)	Hemoglobin (gm/dL)	Potassium (mmol/L)
Sex	MCV (fL)	Chloride (mmol/L)
	MCH (pg)	Bicarbonate (mmol/L)
	MCHC (gm/dL)	Phosphorus (mg/dL)
	RDW (%)	Calcium (mg/dL)
	White blood cells (/𝜇L)	BUN (mg/dL)
	Band neutrophils (/𝜇L)	Creatinine (mg/dL)
	Neutrophils (/𝜇L)	Bilirubin (mg/dL)
	Lymphocytes (/𝜇L)	Glucose (mg/dL)
	Monocytes (/𝜇L)	Total protein (g/dL)
	Eosinophils (/𝜇L)	Albumin (g/dL)
	Basophils (/𝜇L)	Globulin (g/dL)
	Platelets (/𝜇L)	ALT (IU/L)
	MPV (fL)	AST (IU/L)
		ALP (IU/L)
		GGT (IU/L)
		Cholesterol (mg/dL)

Alanine Transaminase (ALT), Aspartate Transaminase (AST), Alkaline Phosphatase (ALP), Blood Urea Nitrogen (BUN), Gamma-glutamyl Transferase (GGT), Mean Corpuscular Hemoglobin (MCH), Mean Corpuscular Hemoglobin Concentration (MCHC), Mean Corpuscular Volume (MCV), Mean Platelet Volume (MPV), Red Blood Cell Distribution Width (RDW).

### Machine learning model performance evaluation and statistical analysis

The 30% test set was used to evaluate MLM performance. Model prediction results are reported as sensitivity and specificity compared to the labeled classification. Receiver operator characteristic (ROC) plots were generated, and the area under the curve (AUC) was calculated. For the PSS SubCat model, a one-versus-rest multiclass ROC was utilized. The 95% confidence intervals (95% CI) of sensitivity, specificity, and AUC were calculated using the Wilson-Brown method (Prism v.9.2.0; GraphPad). Population characteristics were tested for normality. They did not meet normality criteria, and are presented as the median and interquartile range. Comparisons between continuous clinicopathologic variables were performed with a Wilcoxon-Rank Sum test, and *p*-values were adjusted for multiple comparisons using a Bonferroni correction.

Feature importance in the PSS MLM was assessed using the gain metric. Features with a high score were considered to have higher gain and, therefore, more impact on the MLM prediction.

## Results

### Study population demographics

In the PSS group, 1,149 dogs were assessed for eligibility. Of these, 274 were excluded because of a lack of definitive diagnosis of a PSS. From the remaining 875 cases, 223 were excluded for lacking one or both of CBC and serum biochemistry tests. A total of 652 dogs with PSS were included. Of these dogs, 421 had a single extrahepatic shunt, 175 had a single intrahepatic shunt, and 56 had multiple extrahepatic shunts. Table 2 summarizes the method of PSS diagnosis for dogs in this group.

**Table 2:** Method of PSS status determination.

Diagnosis methodology	Computed tomography	Nuclear scintigraphy	Ultrasound	Surgical exploration
PSS Dogs (*n* = 652)	265	290	452	481
Single intrahepatic (*n* = 175)	130	51	110	148
Single extrahepatic (*n* = 421)	120	221	304	329
Multiple extrahepatic shunts (*n* = 56)	15	18	38	4
Non-PSS Dogs (*n* = 589)	1	66	573	1

In the non-PSS group, 969 dogs were assessed for eligibility. One hundred and fifty were excluded for lack of confident exclusion of a portosystemic shunt. A further 230 were excluded for lacking one or both of CBC and serum biochemistry tests, leaving 589 control dogs for analysis. Dogs in the control group had a PSS ruled out by nuclear scintigraphy in 66, computed tomography in 1, and the remaining 522 dogs had a PSS ruled out with abdominal ultrasound. Some dogs had multiple diagnostics performed (Table 2). Within the non-PSS group, the category of clinical diagnosis consisted of 228 dogs with GI disease, 171 with neurologic disease, 54 with neoplasia, 35 with genitourinary disease, 32 with immune-mediated disease, 12 with respiratory disease, 9 with infectious, 7 with cardiac disease, 1 with primary surgical disease, and 40 with an undetermined disease process. Seven of the dogs with GI diseases were diagnosed with PVH after a review of the histopathology of liver biopsy.

The breed group distribution was significantly different between the PSS and control group (*p* < 0.0001), with more toy breed dogs represented in the PSS group (Table 3). Dogs in the PSS group were younger (*p* < 0.0001) and had a smaller body weight (*p* < 0.0001) compared to control dogs. There was no difference in sex distribution between groups (Table 3). Weight measurements were missing for 26 (4.3%) PSS dogs and 21 (9.2%) non-PSS dogs. Age was missing for 4 (0.7%) PSS dogs and 21 (3.6%) non-PSS dogs.

**Table 3:** Demographic description of dogs with and without PSS.

	PSS dogs	Non-PSS dogs
**Sex (*n*, %)**		
Female	338 (52%)	319 (54%)
Male	313 (48%)	268 (46%)
**Breed group (*n*, %)**		
Herding	41 (6%)	57 (10%)
Hound	28 (4%)	39 (7%)
Non-sporting	35 (5%)	81 (14%)
Other	114 (17%)	77 (13%)
Sporting	86 (13%)	88 (15%)
Terrier	50 (8%)	64 (11%)
Toy	265 (41%)	128 (22%)
Working	33 (5%)	55 (9%)
Age (years; median, IQR)	1.5 (0.6–3.9)	6.5 (2.3–10)
Weight (kg; median, IQR)	6.0 (2.9–13)	12 (5.3–27)

### Clinicopathologic findings

Clinicopathologic differences between dogs with PSS and non-PSS control dogs are summarized in Table 4. Individual clinicopathologic values that were missing in >2% of cases included total calcium missing in 274 dogs (22.1%) and red blood cell distribution width in 47 dogs (3.8%).

**Table 4:** Clinicopathologic findings of dogs with and without PSS.

	Reference interval	PSS cases median (IQR)	Non-PSS controls median (IQR)	Adjusted *p* value
**Complete blood count**				
Red blood cells (/𝜇L)	5.6–8	6.6 (5.8–7.2)	6.1 (5.2–6.8)	0.0035
Hemoglobin (gm/dL)	14–19	13 (11–15)	14 (12–16)	0.0035
Hematocrit (%)	40–55	40 (35–45)	41 (35–46)	0.6335
MCV (fL)	65–75	62 (58–65)	68 (65–71)	0.0035
MCH (pg)	22–26	20 (19–22)	24 (23–25)	0.0035
MCHC (gm/dL)	33–36	33 (32–34)	35 (34–36)	0.0035
RDW (%)	11–14	14 (13–15)	14 (13–16)	1
White blood cells (/𝜇L)	6,000–13,000	13,175 (10,298–17,248)	11,000 (8,105–15,770)	0.0035
Band neutrophils (/𝜇L)	0	0 (0–0)	0 (0–0)	0.0105
Neutrophils (/𝜇L)	3,000–10,500	8,393 (6,162–11,908)	7,694 (5,317–12,317)	0.203
Lymphocytes (/𝜇L)	1,000–4,000	2,954 (2,066–3,907)	1,680 (1,044–2,404)	0.0035
Monocytes (/𝜇L)	150–1,200	692 (460–1,173)	600 (380–944)	0.0035
Eosinophils (/𝜇L)	0–1,500	501 (265–847)	276 (104–545)	0.0035
Basophils (/𝜇L)	0–50	23 (0–54)	0 (0–33)	0.0035
Platelets (/𝜇L)	150,000–400,000	238,000 (179,000–308,000)	320,000 (232,250–428,000)	0.0035
MPV (fL)	7–13	9.9 (8.8–11)	9.8 (8.7–12)	1
**Serum chemistry panel**				
Anion gap (mmol/L)	12–20	17 (14–20)	21 (19–24)	0.0035
Sodium (mmol/L)	143–151	147 (145–149)	147 (145–149)	1
Potassium (mmol/L)	3.6–4.8	4.4 (4.1–4.7)	4.5 (4.2–4.9)	0.0035
Chloride (mmol/L)	108–116	113 (110–116)	110 (107–113)	0.0035
Bicarbonate (mmol/L)	20–29	21 (19–23)	21 (19–23)	0.3325
Phosphorus (mg/dL)	2.6–5.2	4.9 (4–6.2)	4.4 (3.6–5.2)	0.0035
Calcium (mg/dL)	9.6–11.2	9.8 (9.3–10)	10 (9.7–11)	0.0035
BUN (mg/dL)	11–33	6 (5–10)	16 (10–22)	0.0035
Creatinine (mg/dL)	0.8–1.5	0.4 (0.3–0.5)	0.7 (0.6–0.9)	0.0035
Glucose (mg/dL)	86–118	94 (82–107)	103 (93–113)	0.0035
Total protein (g/dL)	5.4–6.9	5.1 (4.5–5.7)	6.1 (5.5–6.7)	0.0035
Albumin (g/dL)	3.4–4.3	2.7 (2.4–3)	2.9 (2.4–3.3)	0.0035
Globulin (g/dL)	1.7–3.1	2.4 (1.9–2.8)	3.2 (2.6–3.7)	0.0035
ALT (IU/L)	21–72	114 (60–230)	64 (36–148)	0.0035
AST (IU/L)	20–49	68 (45–117)	36 (26–61)	0.0035
ALP (IU/L)	14–91	150 (81–249)	105 (48–288)	0.0035
GGT (IU/L)	0–5	4 (2–6)	5 (3–8)	0.0035
Cholesterol (mg/dL)	139–353	134 (99–185)	214 (154–276)	0.0035
Bilirubin (mg/dL)	0.0–0.2	0.2 (0.1–0.2)	0.2 (0.1–0.3)	0.2205

*p* values adjusted with Bonferroni-Dunn method, and adjusted values < 0.05 represent significant differences. Alanine Transaminase (ALT), Aspartate Transaminase (AST), Alkaline Phosphatase (ALP), Blood Urea Nitrogen (BUN), Gamma-glutamyl Transferase (GGT), Mean Corpuscular Hemoglobin (MCH), Mean Corpuscular Hemoglobin Concentration (MCHC), Mean Corpuscular Volume (MCV), Mean Platelet Volume (MPV), Red Blood Cell Distribution Width (RDW).

### PSS prediction

**Figure 1:**
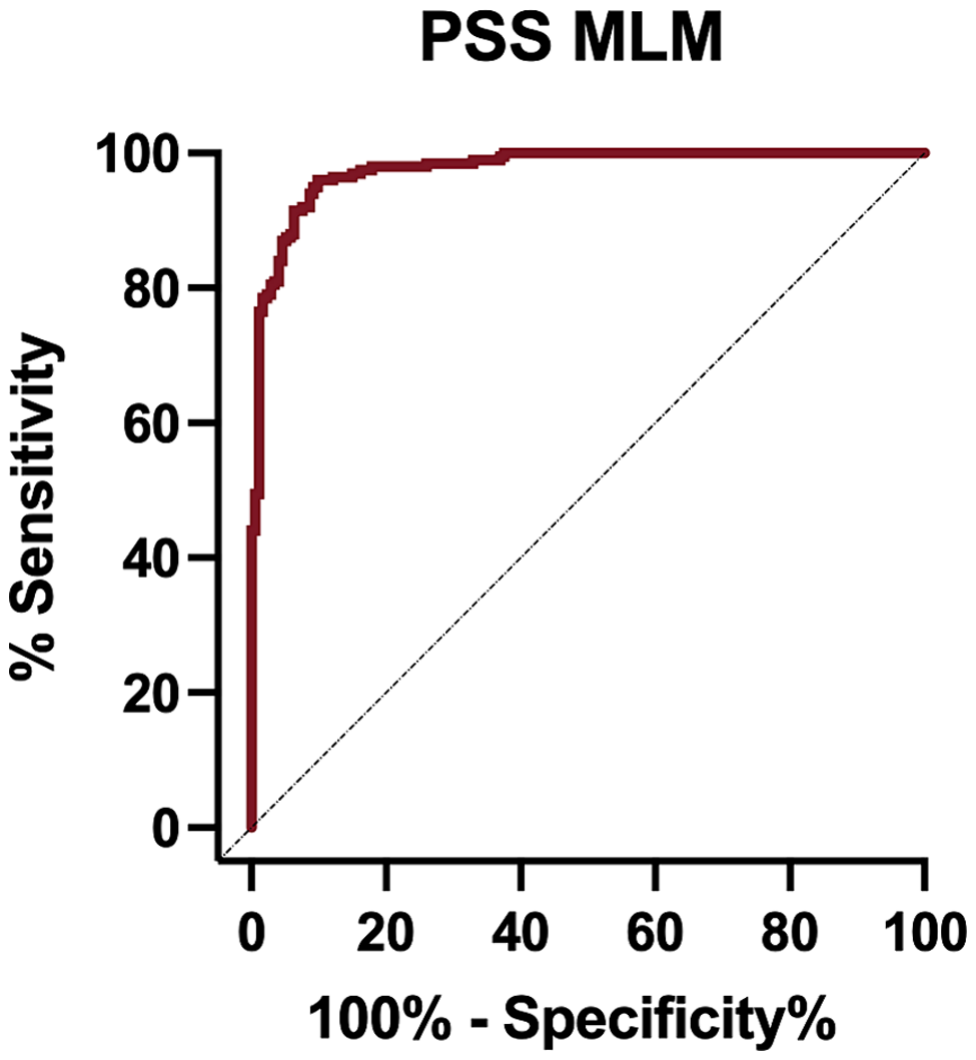
Receiver operator characteristic (ROC) curve for the portosystemic shunt machine learning model on the test set data.

**Figure 2:**
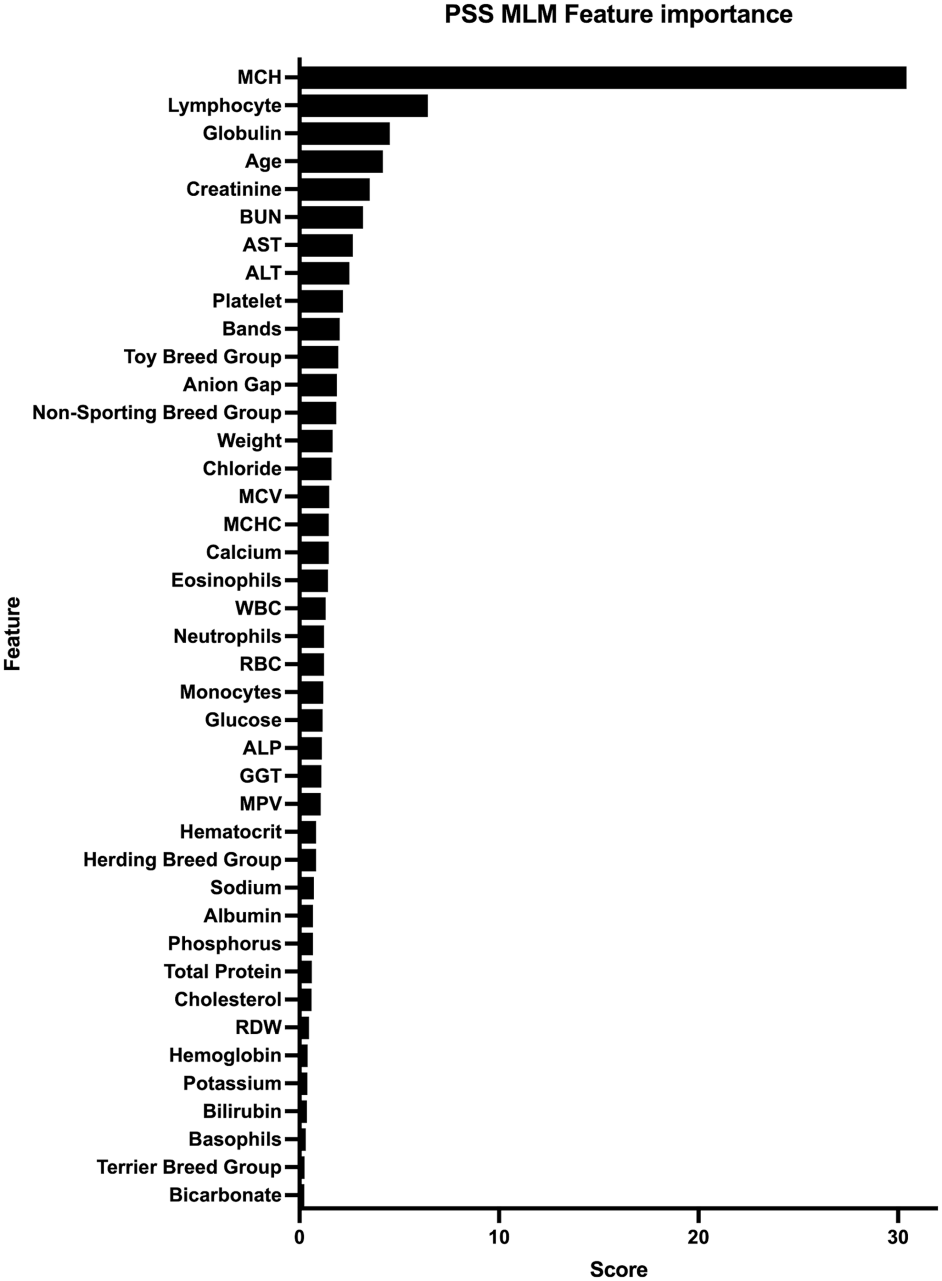
Feature importance for machine learning model. All features used for model training and the corresponding score that represents the gain and importance in model prediction are listed from those with highest importance to those with the lowest importance. Alanine Transaminase (ALT), Aspartate Transaminase (AST), Alkaline Phosphatase (ALP), Blood Urea Nitrogen (BUN), Gamma-glutamyl Transferase (GGT), Mean Corpuscular Hemoglobin (MCH), Mean Corpuscular Hemoglobin Concentration (MCHC), Mean Corpuscular Volume (MCV), Mean Platelet Volume (MPV), Red Blood Cell Distribution Width (%).

The PSS MLM classification model predicted a diagnosis of a PSS correctly in 345/373 (92.5%) dogs in the test set, correctly classifying 183/194 dogs within the PSS group, yielding a sensitivity of 94.3% (95% CI 90.1–96.8%) (Table 5). The classifier correctly classified 162/179 of the non-PSS dogs as not having a PSS, resulting in a specificity of 90.5% (95% CI 85.32–94.0%). The positive and negative likelihood ratios are 9.9 and 0.07, respectively. This model has an AUC of 0.976 (95% CI; 0.964–0.989) (Figure 1). Of the features utilized to train the PSS classification model, MCH, lymphocyte count, and serum globulin concentration were identified as the most important predictors of classification (Figure 2).

**Table 5:** Machine learning model prediction compared to the ground truth of dogs in the test set.

		Ground truth classification	
		PSS dogs	Non-PSS dogs
PSS MLM prediction	PSS predicted	183	17
	No PSS predicted	11	162

### PSS subcategory prediction

**Figure 3:**
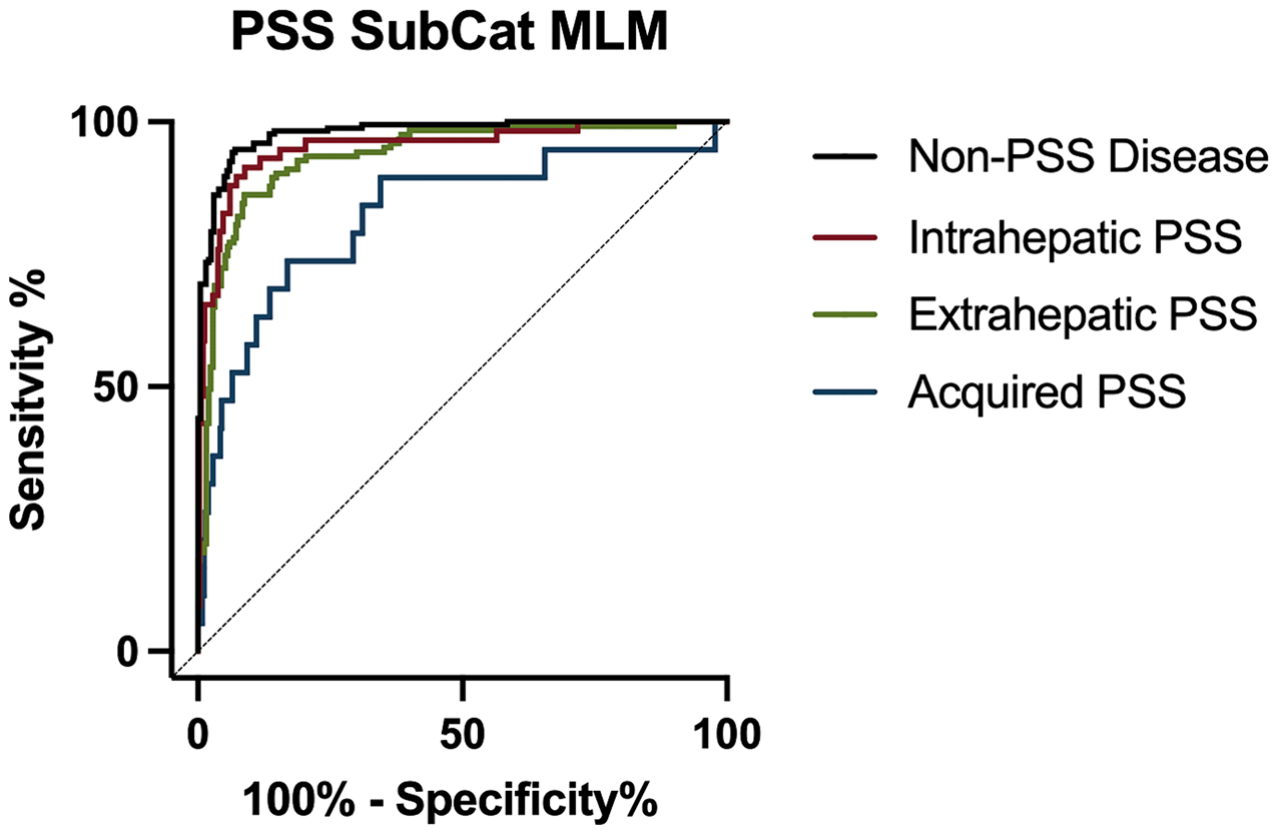
Receiver operator characteristic (ROC) curve for detection of portosystemic shunt (PSS) subcategory classification for intrahepatic PSS (red line), extrahepatic PSS (green line), acquired PSS (blue line), and non-PSS disease (black line).

The PSS SubCat MLM correctly predicted the classification of 7/13 (33.9%) of dogs with acquired PSS, 103/121 (85.1%) of dogs with extrahepatic PSS, 46/58 (79.3%) of dogs with intrahepatic PSS, and 164/181 (90.6%) of dogs with non-PSS disease with an overall accuracy of 85.7% (Table 6). When assessing one subcategory versus all other categories, this model has an AUC of 0.954 (95% CI; 0.921–0.986) for detecting intrahepatic PSS, 0.937 (95% CI; 0.910–0.963) for detecting extrahepatic PSS, 0.824 (95% CI; 0.711–0.938) for detecting acquired PSS, and 0.979 (95% CI; 0.967–0.991) for detecting non-PSS disease (Figure 3; Table 7).

**Table 6:** Four by four table demonstrating PSS SubCat MLM prediction compared to ground truth classification on the test set.

		**Ground truth classification**			
		Acquired PSS	Extrahepatic PSS	Intrahepatic PSS	Non-PSS disease
PSS SubCat MLM prediction	Acquired PSS	7	6	0	6
	Extrahepatic PSS	2	103	8	10
	Intrahepatic PSS	2	9	46	1
	Non-PSS disease	2	3	4	164

**Table 7:** The performance of PSS SubCat MLM on the test set.

Subcategory	Sensitivity (%)	Specificity (%)	AUC
Acquired PSS	53.9 (29.1–76.8)	96.7 (94.3–98.1)	0.824 (0.711–0.938)
Intrahepatic PSS	79.3 (67.2–87.8)	96.2 (93.5–97.8)	0.954 (0.921–0.986)
Extrahepatic PSS	85.1 (77.7–90.4)	92.1 (88.0–94.8)	0.937 (0.910–0.963)
Non-PSS disease	90.6 (85.5–94.0)	95.3 (91.3–97.5)	0.979 (0.967–0.991)

Numbers in parentheses are 95% CIs.

## Discussion

MLMs trained with routinely collected patient data can accurately predict the presence and subtype of PSS. These MLMs can identify patterns with dog signalment, hematologic, and biochemical parameters that differentiate between PSS and non-PSS disease with an accuracy similar to traditional methods of PSS detection, including ultrasonography, serum post-prandial bile acids, and plasma ammonia concentrations (8, 12, 24). Application of these MLMs may provide actionable diagnostic information utilizing readily available laboratory parameters that can augment clinical decision-making.

Measurement of post-prandial serum bile acids or plasma ammonia concentrations are commonly used biomarkers to screen for PSS in dogs. Post-prandial serum bile acids are a sensitive marker of PSS (92–93%), but specificity (67%) is lacking (8, 24). Elevated plasma ammonia concentrations have a sensitivity of 85–100% and a specificity of 84–89% (8, 24). Limitations to applications of these assays include the need for a 12 h fast before performing a bile acids test, the liable nature of ammonia, the need for reference laboratory testing, and the associated costs. Compared to these biomarkers, the MLM trained to detect the presence or absence of a PSS has equivalent sensitivity (94%) with superior specificity (90%), without the practical limitations of biomarker testing. Further, serum bile acids and plasma ammonia concentration testing cannot differentiate between subcategories of PSS. In contrast, a second MLM was trained to predict the subcategory of PSS and had an overall accuracy of 86%. This study did not include a comparison of serum bile acid concentrations, plasma ammonia concentrations, and MLM performance, as many of the PSS dogs did not have this biomarker testing performed at our institution.

Feature importance for the MLM was determined in this study. Understanding the most contributive features allows for model transparency, confidence during clinical decision-making, and may expose physiologic features of PSS that were not previously recognized (25). The most contributive features in this study were MCH, lymphocyte count, and serum globulin concentrations.

The MCH is a calculated value representing the hemoglobin amount per red blood cell. A low MCH has been previously associated with the presence of PSS (26, 27). It has been suggested that this may be due to impaired iron utilization and abnormal iron metabolism in dogs with PSS. MCH increases and decreases proportionally to cell volume, and indeed dogs with PSS in this group had lower MCV than non-PSS dogs; however, MCH was more strongly associated with the presence or absence of PSS than MCV. This indicates that MCH is impacted by factors beyond cell size in dogs with PSS. In people, MCH is an independent predictor of iron deficiency, length of hospital stay of people with acute pancreatitis, and premature discontinuation of antiplatelet therapy in people receiving cardiovascular stents indicating this hematologic parameter provides additional information to the more traditionally utilized hemoglobin and MCV (28–30).

Dogs with PSS in our study had higher lymphocyte counts than dogs without PSS. The mechanism of this difference is unknown, but normal to high lymphocytes have been noted previously in dogs and cats with PSS (31, 32). This may reflect age, as the PSS group was younger than the non-PSS group, and lymphocyte counts decrease with age (33). Similarly, serum globulin concentration was associated with PSS status, but may reflect changes in age in the population as globulin concentrations increase with age (34). However, these findings were independent of age in our model, indicating other mechanisms may be contributing.

The MLMs presented here were trained on dogs with a high pre-test probability of a PSS. All dogs in non-PSS group had post-prandial bile acid concentrations determined, signifying the attending clinician suspected hepatic insufficiency, PSS, or some other hepatic disease. Therefore, the application of these MLMs would only be appropriate for dogs with a high suspicion of PSS rather than as a screening tool broadly applied to a population with a low pre-test probability of a PSS.

This study’s control population included dogs tested for a PSS with post-prandial serum bile acid concentrations but had the disease ruled out with at least one imaging modality. This includes many dogs that had a PSS ruled out using an abdominal ultrasound rather than the gold standard, nuclear scintigraphy. The sensitivity and specificities for the detection of PSS with abdominal ultrasound varies widely, ranging from 47 to 85% and 67 to 100%, respectively (6, 12, 13, 35). In our institution, highly skilled ultrasonographers interpreting the study, and the holistic assessment of the patient by the attending clinician refuted the presence of a PSS. However, a limitation of this study is the lack of definitive diagnosis in many non-PSS dogs. Additionally, the non-PSS dogs represent at heterogenous group of disease processes as demonstrated by the varied categories of disease in this group, but was predomidated by dogs with primary gastrointestinal disease or neurologic disease. This highlights the ability of this model to identify dogs with PSS in dogs with varied clinical presentations.

A further limitation of this study is the unbalanced data set and the limited number of dogs with some subsets of PSS. Most dogs with PSS were dogs with a single, extrahepatic PSS, followed by dogs with a single, intrahepatic PSS, and the fewest number with multiple, acquired, extrahepatic shunts, consistent with the epidemiology of the disease (20, 36). To address this, a method of over-sampling categories with fewer dogs was utilized that can enrich the data set (22, 23). Future studies should include larger groups of dogs with acquired, extrahepatic shunts to enrich the training set used to subcategorize types of PSS. These MLMs are trained with blood work performed from a single clinical laboratory over a 20 year time period. This represents a wide period of time necessary to amass the patient numbers necessary to train MLM, yet also introduces limitations, including variations in clinicopathologic methodologies over this period. Indeed we noted that some dogs had missing clinicopathologic variables that were not included on the blood work panels during some time periods. However, we utilized models in this study designed to handle missing values in features without impacting performance (37, 38). Future studies should include clinicopathologic data from multiple clinical laboratories with varied instrumentation to determine the generalizability of these models.

The project adopts an open-source framework to facilitate the integration of this method into clinical workflows. This design choice promotes transparency and allows collaborative enhancements, where clinicians and developers can explore the codebase. The proposed non-invasive diagnosis solution could be integrated into electronic medical record systems, allowing clinicians to utilize these clinical decision-support tools in real time.

In conclusion, MLMs can provide accurate prediction of the presence of PSS and subcategories of disease using routinely collected clinicopathologic and signalment data. These MLMs have similar sensitivity to traditional biomarkers used for detecting PSS in dogs with improved specificity.

## Data availability statement

The raw data supporting the conclusions of this article will be made available by the authors, without undue reservation.

## Author contributions

MF: Data curation, Formal analysis, Investigation, Methodology, Validation, Writing – original draft, Writing – review & editing, Software. KR: Data curation, Formal analysis, Investigation, Methodology, Validation, Writing – original draft, Writing – review & editing, Conceptualization, Funding acquisition, Project administration, Supervision. AZ: Conceptualization, Data curation, Formal analysis, Funding acquisition, Investigation, Methodology, Project administration, Supervision, Validation, Writing – original draft, Writing – review & editing.
